# Berberine-loaded polylactic acid nanofiber scaffold as a drug delivery system: The relationship between chemical characteristics, drug-release behavior, and antibacterial efficiency

**DOI:** 10.3762/bjnano.15.7

**Published:** 2024-01-12

**Authors:** Le Thi Le, Hue Thi Nguyen, Liem Thanh Nguyen, Huy Quang Tran, Thuy Thi Thu Nguyen

**Affiliations:** 1 Phenikaa University Nano Institute (PHENA), Phenikaa University, Hanoi 12116, Vietnam; 2 School of Material Science and Technology, Hanoi University of Science and Technology, Hanoi 11600, Vietnam; 3 Faculty of Biomedical Sciences, Phenikaa University, Hanoi 12116, Vietnam

**Keywords:** antibacterial activity, berberine, drug-release system, electrospun nanofiber, polylactic acid

## Abstract

Hydrophobic berberine powder (BBR) and hydrophilic BBR nanoparticles (BBR NPs) were loaded into an electrospun polylactic acid (PLA) nanofiber scaffold for modulating the release behavior of BBR in an aqueous medium. The BBR release from the BBR/PLA and BBR NPs/PLA nanofiber scaffolds was investigated in relation to their chemical characteristics, BBR dispersion into nanofibers, and wettability. The BBR release profiles strongly influenced the antibacterial efficiency of the scaffolds over time. When the BBR was loaded, the BBR/PLA nanofiber scaffold exhibited an extremely hydrophobic feature, causing a triphasic release profile in which only 9.8 wt % of the loaded BBR was released in the first 24 h. This resulted in a negligible inhibitory effect against methicillin-resistant *Staphylococcus aureus* bacteria. Meanwhile, the BBR NPs/PLA nanofiber scaffold had more wettability and higher concentration of BBR NPs dispersed on the surface of PLA nanofibers. This led to a sustained release of 75 wt % of the loaded BBR during the first 24 h, and consequently boosted the antibacterial effectiveness. Moreover, the cytotoxicity test revealed that the BBR NPs/PLA nanofiber scaffold did not induce any changes in morphology and proliferation of MA-104 cell monolayers. It suggests that the BBR/PLA and BBR NPs/PLA nanofiber scaffolds can be used in different biomedical applications, such as wound dressing, drug delivery systems, and tissue engineering, according to the requirement of BBR concentration for the desired therapeutic effects.

## Introduction

Medicinal plants have various biologically active compounds, such as phenolic acids, alkaloids, saponins, coumarins, flavonoids, terpenoids, and carotenoids with great therapeutic effects [1]. Berberine (BBR) is a quaternary isoquinoline alkaloid, extracted from different medicinal plants, such as *Coptis chinensis* and *Berberis vulgaris* [2], and used in the treatment of central nervous system disorders [2], digestive system diseases [3], cancer, diabetes, inflammation, and infections. Nevertheless, BBR has a low bioavailability due to its poor water solubility, which imposes a regular intake of BBR drugs at a high dose. Recently, innovative technologies have been employed to produce nanoformulations of drugs for endowing a better therapeutic effect. The nanoformulations for drug delivery can be designed using nanocarrier systems, including organic materials (liposomes, nanoemulsions, nanomicelles, and nanofibers) and inorganic nanoparticles (gold, silver, iron oxide, and mesoporous silica nanoparticles) [4]. Additionally, nanocarrier-free systems, such as drug nanocrystals, are also used to improve the delivery of poorly soluble drugs [5–6]. In our previous study, the saturation concentration of BBR in water was 2.0 mg/mL, while BBR nanoparticles prepared by antisolvent precipitation could reach up to 5.0 mg/mL, which notably increased the antibacterial activity of BBR [7].

Electrospinning is a convenient technique that allows one to fabricate nanofiber scaffolds with various compositions and structures. During the electrospinning process, a polymer solution blended with additional components is applied under a high-voltage electrostatic field, generating a charged and stretched solution jet following nanofiber formation [8–9]. Drug delivery systems based on nanofiber scaffolds produced by electrospinning method have strongly attracted researchers due to their unique characteristics. First, high porosity and large surface-to-volume ratio of nanofiber scaffolds give the material the potential to be exposed to the biological media for drug release. Besides, 3D nanofiber scaffolds resemble the natural extracellular matrix, promoting nutrients and cells to penetrate into their structure [10]. Second, high drug loading can be achieved, and the drug-release profile (i.e., prolonged, stimulus-activated, and biphasic releases) can be modulated by using different nanofiber structures (e.g., blending, core/shell, and multilayer structures) and nanofiber compositions [11–13]. For a long-term drug release, hydrophobic polymers are chosen for the preparation of drug-loaded nanofiber scaffolds. This is because the hydrophobicity of the polymer could form air gaps, slowing matrix hydration and suppressing drug diffusion from the nanofibers [14]. The core/shell nanofiber structure can also prolong the drug release since the polymer shell plays a role as a rate-control barrier [15]. On the other hand, the nanofiber scaffolds fabricated using suitable hydrophilic or water-soluble polymers are used to improve the dissolution profile and bioavailability of poorly soluble drugs [15]. Limoee et al. reported that the release rate of Pramipexole from hybrid cross-linked nanofibers was successfully controlled between 8 and 10 h, which is approximately the same time that the drug formulation travels from the mouth to the small intestine. It is worthwhile mentioning that the hybrid cross-linked nanofibers in this work were made of a mixture of a hydrophilic polymeric matrix (polyvinyl alcohol and carboxymethyl cellulose) and a hydrophobic polymer (polycaprolactone) [16]. Interestingly, the nature of the drug and the drug–polymer compatibility strongly affect the release behavior of the drug from nanofiber scaffolds [14,17–19]. Polylactic acid (PLA), a synthetic polymer that has been approved by the FDA for biomedical usage, is commonly used for drug delivery systems due to its biocompatibility and biodegradability [20]. Yuan et al. [17] explained the difference in the drug-release profile of PLA nanofibers loaded with hydrophilic doxorubicin hydrochloride (Dox-HCl) and hydrophobic free base doxorubicin (Dox-base). The rapid release of Dox-HCl from the PLA nanofiber carrier was attributed to Dox-HCl crystal aggregates mainly distributed on the surface of the fibers. In addition, the reduction in the hydrophobicity of the nanofiber network also caused a faster release of the drug. Meanwhile, the hydrophobic PLA nanofiber carrier showed a sustained release behavior of the Dox-base. This was because hydrophobic Dox-base significantly improved the miscibility with the PLA, forming a uniform drug dispersion in the PLA matrix and, therefore, restricting the drug release.

One of the main issues in clinical treatments is bacterial infections, which prolong treatment time or cause further complications. Among various types of nanomaterials, nanofiber scaffolds can act as a multifunctional tool in medical treatments, combining drug release for disease therapy, cell proliferation, wound healing, and antimicrobial effect [21–25]. Nanofibers of PLA functionalized with laponite (LAP)/amoxicillin (AMX) prolonged the drug release up to 21 days and inhibited the growth of *Staphylococcus aureus* and *Escherichia coli* bacteria. Human bone marrow mesenchymal stem cells were well attached and proliferated on the surface of the LAP/AMX functionalized PLA scaffolds, which provided a bacteria-free environment for bone differentiation in the treatment of bone defects [21]. In dentistry, anti-infective nanofiber-based drug-release systems have been investigated for periodontal disease control, endodontic therapy, cariogenic microorganism control, and tissue reconstruction [25]. Due to the controlled drug release, BBR-loaded nanofiber scaffolds exhibited excellent performance in repairing bone defects [3,26], healing diabetic foot ulcers [27], promoting hemostasis [28], acting as anti-leishmanial drugs [29], and inhibiting microbial agents [27,30]. Zhou et al. [31] developed hybrids of nanofibers and microparticles for dual-step controlled release of BBR, combining a fast-release step of BBR from hydrophilic polypyrrolidone nanofibers (47.9 wt % in the first hour) and a sustained-release step of BBR from the insoluble cellulose acetate microparticles (98.6 wt % for 60 h). In comparison with the aforementioned hybrid nanofibers, the release rate of BBR from PCL nanofibers [28] was significantly lower with an initial BBR release of 38 wt % in the first day, and a subsequent sustained BBR release of 76% during seven days. Meanwhile, a lower burst release of BBR from PCL/collagen nanofibers [3] was achieved on the first day (14.83 wt %) and this scaffold could prolong the release of BBR up to 27 days (81.4 wt %). The difference in BBR release profiles of these nanofiber scaffolds can be attributed to the difference in chemical characteristics of the polymer matrix, the content of BBR in the nanofibers, and the morphology of the nanofibers.

The release profile of antimicrobial agents should be adjusted depending on the infection conditions, such that a burst release mode is required for acute microbial infections, while slow and long-term release is more adequate to treat chronic infections [32]. This study aims to investigate the drug-release behavior of BBR from the electrospun PLA nanofiber scaffold, regarding drug–polymer compatibility and hydrophobicity of the scaffold. Besides, the antibacterial activity of these scaffolds relating to the release of BBR during 24 h was examined against methicillin-resistant *Staphylococcus aureus* (MRSA). The PLA nanofiber scaffold loaded with hydrophilic BBR nanoparticles showed faster BBR release compared with that of the hydrophobic BBR powder-loaded scaffold, resulting in better inhibitory effects against MRSA. The findings of this study suggest controlled drug-release profiles from nanofiber-based drug delivery systems for specific applications.

## Result and Discussion

### Morphology of PLA and BBR-loaded PLA nanofiber scaffolds

**Figure 1:**
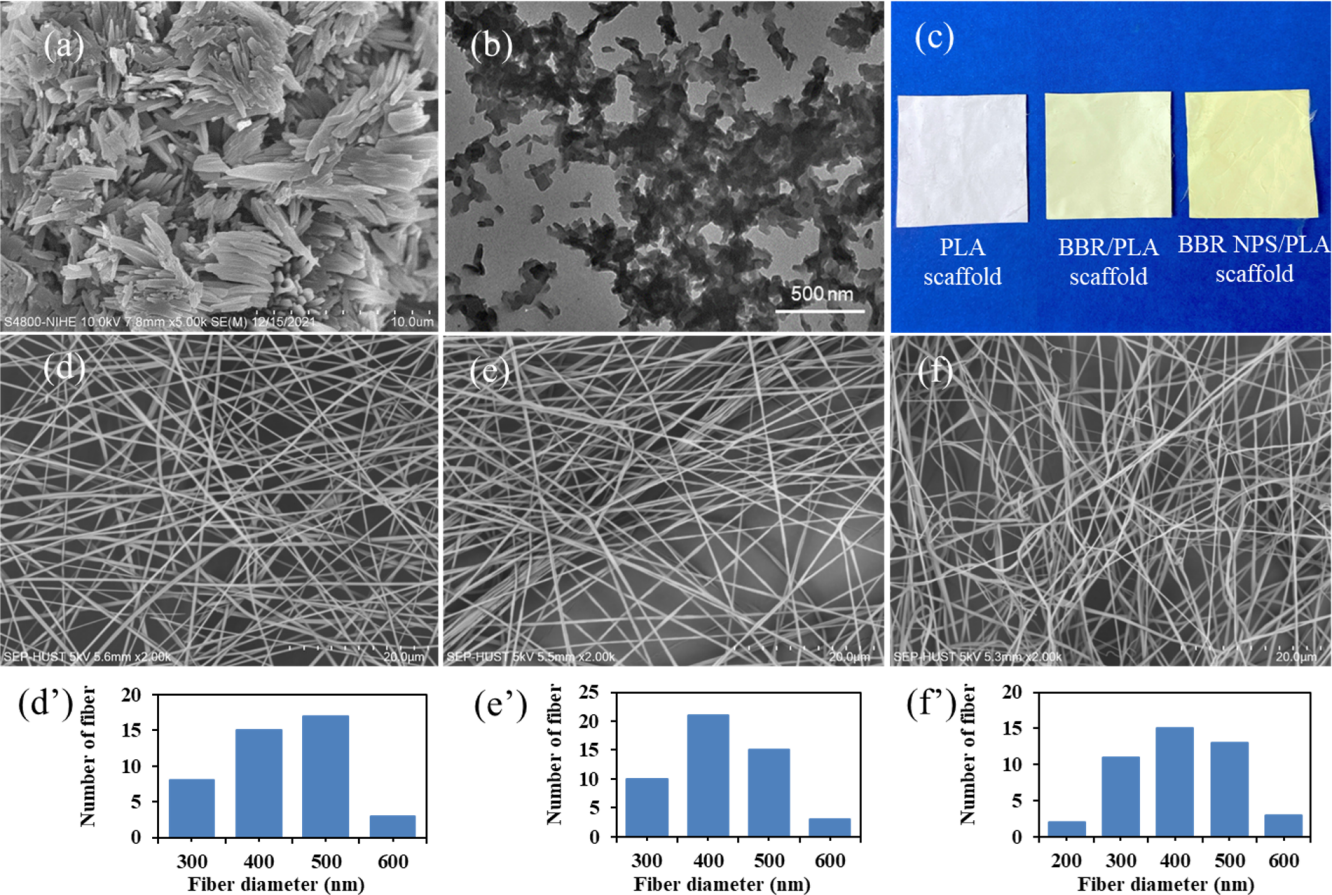
(a) SEM image of BBR powder. (b) TEM image of BBR NPs. (c) Digital image of the electrospun nanofiber scaffolds. (d, d’), (e, e’), (f, f’) SEM images and the distribution of fiber diameter of electrospun PLA, BBR/PLA, and BBR NPs/PLA nanofiber scaffolds, respectively.

In order to evaluate the distribution of BBR compositions in the electrospun PLA nanofibers, the morphology of BBR powder, BBR NPs, and electrospun nanofibers was observed by scanning electron microscopy (SEM, Figure 1). The BBR powder appeared as aggregates of rods in the micrometer size (Figure 1a), while BBR NPs were formed as nanoscale rectangles (Figure 1b). The electrospun PLA nanofibers showed bead-free and uniform morphology with fiber diameter in the range of 200–600 nm. The addition of BBR powder did not strongly affect the morphology of electrospun BBR/PLA nanofibers, except for a slight decrease in the average fiber diameter as shown in Table 1. This indicates that the hydrophobic BBR powder was well dissolved in the hydrophobic PLA polymer in the mixture of dichloromethane and *N*,*N*-dimethylformamide (DCM/DMF) solvent. Meanwhile, electrospun PLA nanofibers incorporated with BBR NPs had a wider diameter distribution with the addition of fiber diameters below 200 nm. Additionally, the BBR NPs/PLA nanofibers were more entangled and less uniform compared with BBR/PLA nanofibers. There is a possibility that BBR NPs were more hydrophilic than the BBR powder, leading to lower compatibility in the hydrophobic PLA polymer [17,33]. The incorporation of the BBR drug in PLA nanofibers resulted in a smaller fiber diameter, which was attributed to the positively charged quaternary ammonium groups of BBR, increasing the charge density of the blend solution. As the higher charged solution jet, the elongation force imposed on the jet was higher, forming smaller fibers [3,34]. Interestingly, although the same amount of BBR drug was incorporated in the BBR/PLA and BBR NPs/PLA electrospun nanofiber scaffolds, the latter appeared with a darker yellow color, which is typical of the natural color of BBR (Figure 1c).

**Table 1:** Average diameter and water contact angle of electrospun PLA, BBR/PLA, and BBR NPs/PLA nanofiber scaffolds.

Type of nanofiber scaffold	Average diameter (nm)	Water contact angle (°)

PLA	395 ± 78	130.1 ± 1.3
BBR/PLA	351 ± 65	126.3 ± 1.6
BBR NPs/PLA	356 ± 98	107.0 ± 2.2

### Chemical characteristics and wettability of BBR-loaded PLA nanofiber scaffolds

**Figure 2:**
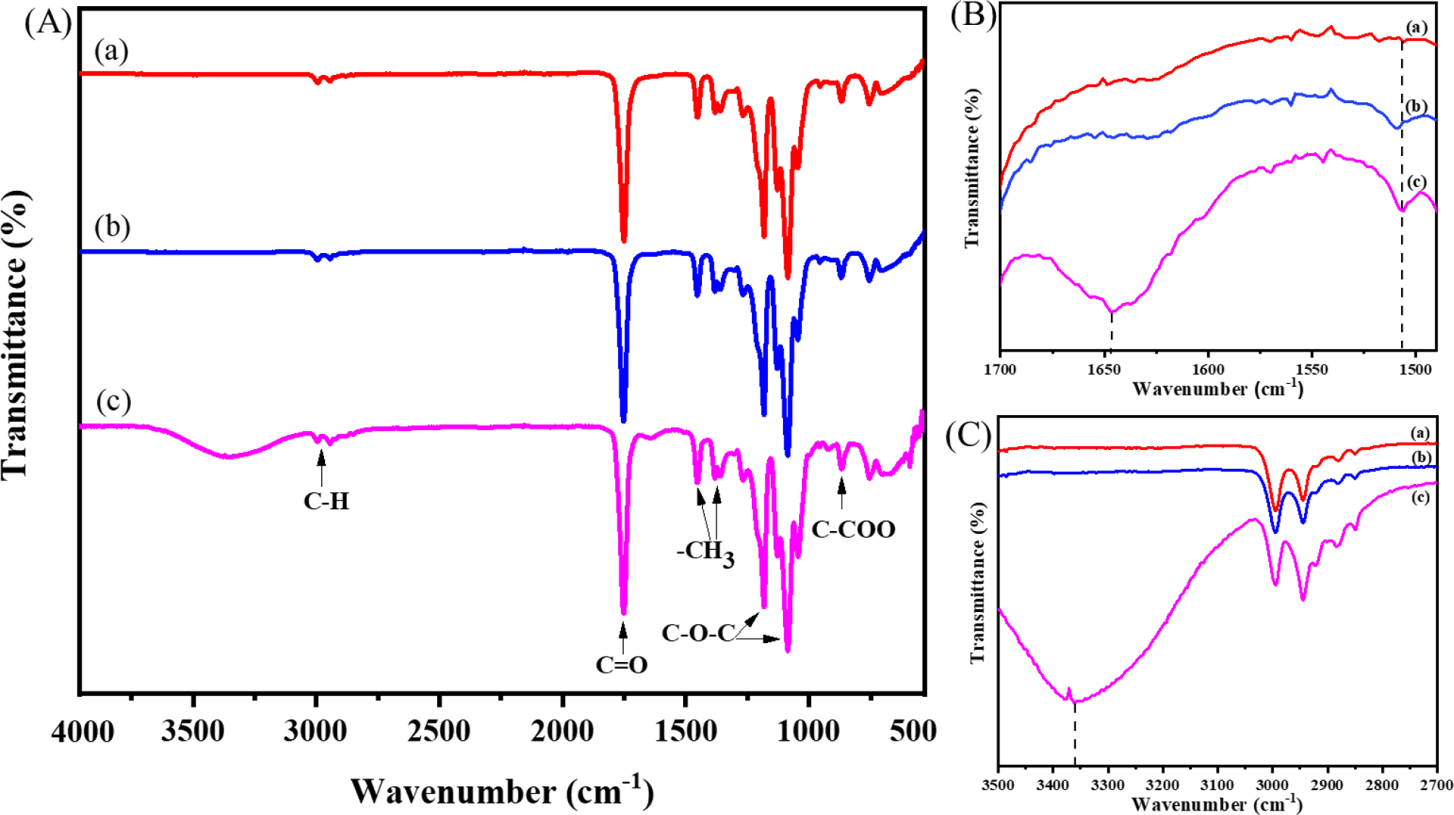
FTIR spectra of (a) PLA, (b) BBR/PLA, and (c) BBR NPs/PLA nanofiber scaffolds at different wavenumber ranges (A, B, C).

By identifying distinct vibrational modes of various chemical bonds, Fourier transform infrared spectroscopy (FTIR) was used to examine the differences in chemical characteristics of BBR-loaded PLA nanofiber scaffolds (Figure 2A). The FTIR spectrum of PLA nanofiber scaffold shows the adsorption peaks at 1751 cm^−1^ resulting from the stretching vibrations of the C=O bond in carboxylic groups. The two bands at 1182 cm^−1^ and 1087 cm^−1^ were attributed to the C–O–C binding vibrations. The absorption bands at 2992 cm^−1^ and 2947 cm^−1^ were characteristics of asymmetrical and symmetrical stretching vibrations of the C–H bond, while the asymmetrical vibrations of –CH_3_ appeared at 1453 and 1358 cm^−1^. These aforementioned adsorption characteristics of PLA nanofiber scaffolds were also observed in the FTIR spectra of BBR/PLA and BBR NPs/PLA nanofiber scaffolds. However, there was the addition of an intense broadband at 3361 cm^−1^ in the FTIR spectrum of the BBR NPs/PLA nanofiber scaffold. This was due to the O–H stretching vibration of the glycerol component in BBR NPs (Figure 2). Peculiarly, the absorption bands at 1646 cm^−1^ and 1506 cm^−1^, characteristic of the C=N^+^ double bond and the furyl group in the molecular structure of BBR, respectively, were only displayed in the FTIR spectrum of the BBR NPs/PLA nanofiber scaffold. This is evidence that the chemical characteristics of BBR on the BBR NPs/PLA nanofibers were detected more clearly than that of the BBR/PLA nanofibers at the same amount of BBR incorporated into nanofibers, possibly due to the higher concentration of the BBR molecule on the surface of BBR NPs/PLA nanofibers. The observation of Figure 1c further supports this assumption.

**Figure 3:**
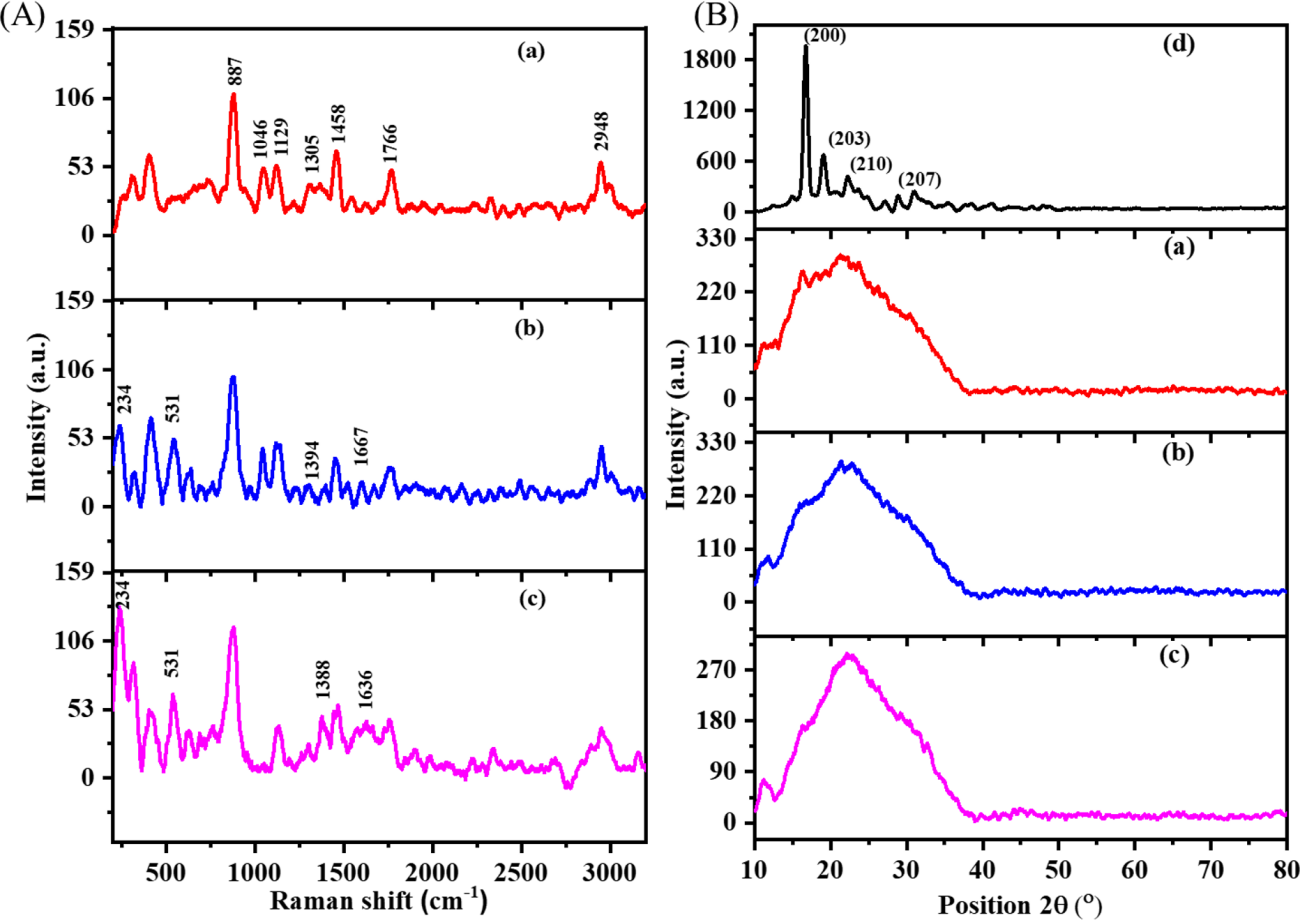
(A) Raman spectra of (a) PLA, (b) BBR/PLA, and (c) BBR NPs/PLA nanofiber scaffolds and (B) XRD patterns of (a) PLA, (b) BBR/PLA, and (c) BBR NPs/PLA nanofiber scaffolds and (d) PLA pellet.

The analysis of Raman spectra (Figure 3A) was employed to confirm chemical characteristics of PLA, BBR/PLA, and BBR NPs/PLA nanofiber scaffolds. The distinct peaks of the PLA nanofiber scaffold were found at 887, 1046, 1129, 1305, 1458, 1766, and 2948 cm^−1^ corresponding to the vibration of υC–COO stretching, υC_α_–C_β_ stretching, *r*CH_3_ rocking, δCH bending, δCH_3_ asymmetric deformation, υC=O stretching, and υCH_3_ stretching modes [35–36]. In the case of BBR drug-loaded PLA nanofiber scaffolds, there were peaks at 234, 531, 1388, and 1636 cm^−1^ characteristic of BBR [7,37]. However, the intensity of the characteristic peaks of BBR loaded in PLA nanofiber scaffold was markedly decreased compared with those of BBR NPs loaded in the PLA nanofiber scaffold. This evidence strongly supports the conclusion that the BBR concentration on the surface of BBR NPs/PLA nanofibers was higher than that on the surface of BBR/PLA nanofibers, which is in agreement with the above FTIR analysis. The poor miscibility between the hydrophilic drug and the hydrophobic polymer might cause phase separation during the electrospinning process [17,38–39], leading to the formation of a BBR-rich phase on the surface of nanofibers.

The crystallinity of the PLA pellet and electrospun nanofiber scaffolds were examined by X-ray diffraction (XRD) analysis (Figure 3B). The XRD pattern of the PLA pellet shows diffraction peaks at 2θ of 16.7, 19.2, and 22.4° associated to a crystalline α-form orthorhombic structure (card number 00-054-1917, Diffract Plus 2005), while the weak diffraction peaks at 28.9 and 30.9° were characteristic of the β-form trigonal structure [40]. The XRD pattern of the PLA nanofibers formed after stretching the PLA solution during the electrospinning process displays only a broad scattering band, located at around 2θ = 21.3°, indicating an amorphous structure of PLA nanofibers. Due to the stretching and rapid solidification of the PLA solution during traveling from the needle to the collector, the rearrangement of the polymer chains into lamellar packing was limited, resulting in domination of the amorphous region in PLA nanofibers [41]. The XRD patterns of BBR drug-loaded PLA nanofiber scaffolds exhibit a distinct peak similar to that of the PLA nanofiber scaffold without the appearance of the characteristic peaks of BBR NPs at 6.79°, 9.13°, and 13.90° as reported in a previous study [42].

The wettability of the drug-loaded nanofiber scaffolds is an important factor affecting their drug-release behavior. It is reported that the high hydrophobicity of drug-loaded nanofibers resulted in prolonged drug release due to the delayed penetration of water into the polymer scaffolds. The change in the water contact angle of PLA nanofiber scaffolds loaded with BBR powder and BBR NPs is presented in Table 1. The PLA nanofiber scaffold possessed typical hydrophobic property with a water contact angle value of 130.1 ± 1.3°. This value was slightly decreased to 126.3 ± 1.6° when the BBR powder was added to the nanofibers. Meanwhile, the water contact angle value of the BBR NPs/PLA nanofiber scaffold was reduced by 23° relative to that of the PLA nanofiber scaffold, attributing to the hydrophilic BBR NPs favorably concentrated on the surface of the nanofibers. The relationship between the wettability and the BBR-release behavior of BBR-loaded PLA nanofiber scaffolds will be reported in the following drug-release profiles.

### In vitro drug-release profiles and release kinetics

**Figure 4:**
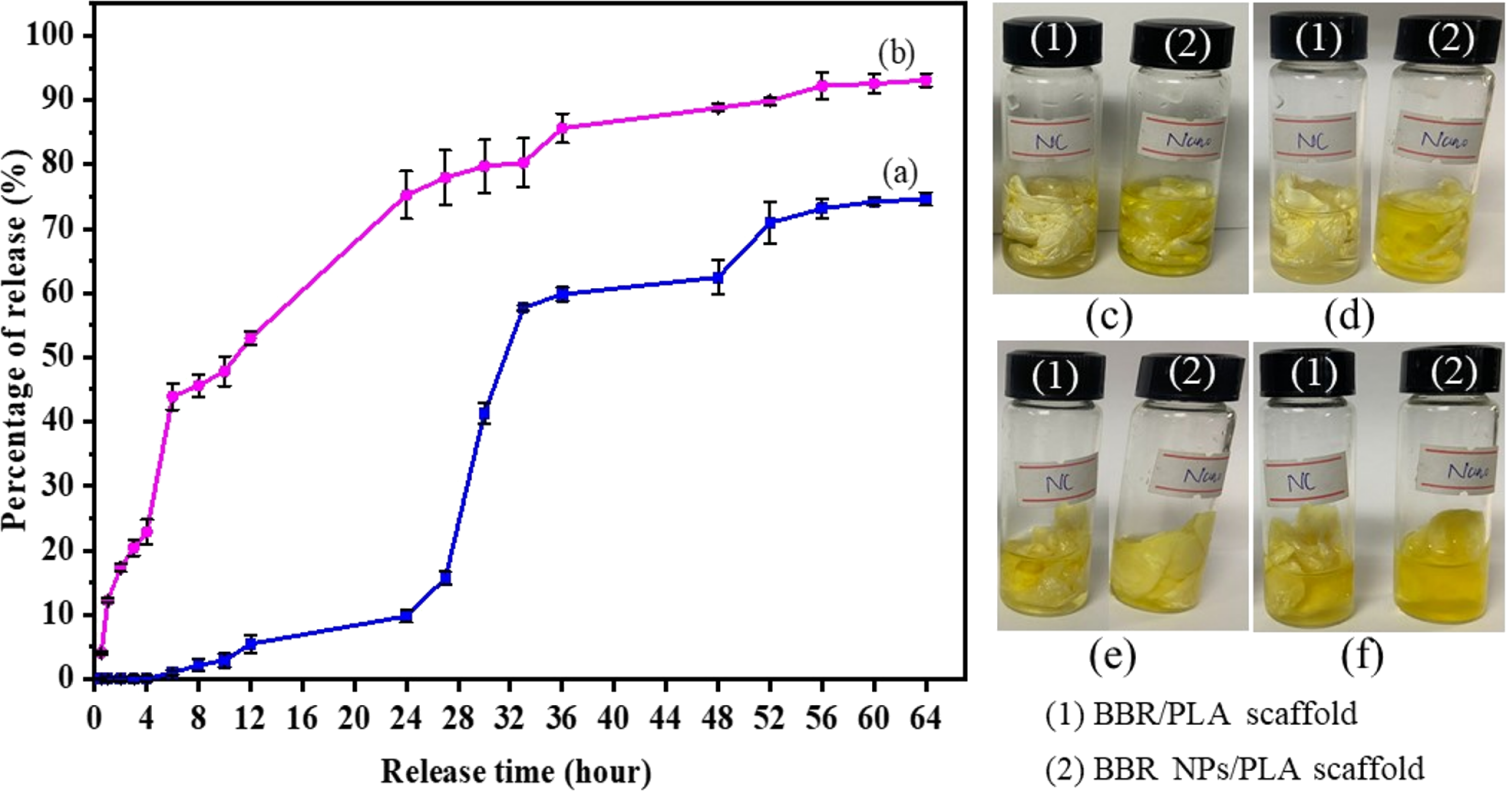
In vitro BBR release profiles of (a) BBR/PLA and (b) BBR NPs/PLA nanofiber scaffolds. (c, d, e, f) Digital images of experimental tests for BBR release for 12, 24, 48, and 56 h.

In vitro release profiles of BBR from BBR/PLA and BBR NPs/PLA nanofiber scaffolds were showed in Figure 4. It can be seen that BBR/PLA and BBR NPs/PLA nanofiber scaffolds exhibited different drug-release characteristics, which were triphasic and sustained BBR-release profiles, respectively. In the case of the BBR/PLA nanofiber scaffold, a slow release of BBR was observed during the first 24 h (lag time), attributed to the hydrophobicity of the scaffold requiring a long time for water permeation. When the scaffold was wetted, BBR was fast released, reaching approximately 60% of the loaded BBR in 36 h. However, in the next 28 h, the BBR/PLA nanofiber scaffold additionally released 15% of the BBR loaded at a slow rate, possibly due to the hardly diffused out BBR embedded in the core region of the nanofibers. As discussed above, the wettability of the BBR NPs/PLA nanofiber scaffold was significantly enhanced. Hence, the BBR was gradually released from the BBR NPs/PLA nanofiber scaffold within 64 h without lag time, and the final release percentage reached a high value of 93%. It is worth mentioning that the release profiles of BBR from BBR/PLA and BBR NPs/PLA nanofiber scaffolds may be suitable for various applications which require different release behaviors for desired therapeutic effects. Ma et al. [3] reported that the prolonged release of BBR from PCL/collagen nanofiber scaffolds up to 27 days was favorable for bone tissue repair. Meanwhile, a high concentration of BBR release within the first 24 h brought good antibacterial activity for wound dressing [28,30].

In order to study the mechanism of BBR release from BBR/PLA and BBR NPs/PLA nanofiber scaffolds, the release data were fitted to several kinetic models, including zero-order, first-order, Higuchi, and Ritger–Peppas models. The regression equations and parameters determined by fitting the BBR release data to the aforementioned mathematical models are shown in Supporting Information File 1 and Table 2. The correlation coefficient (*R*^2^) of BBR release from the BBR/PLA nanofiber scaffold was the largest when fitted to the Ritger–Peppas model compared to that of other models. In addition, the release exponent (*n*) of the equation was 0.1703, indicating that the BBR release from the BBR/PLA nanofiber scaffold followed the Fickian diffusion. In this mechanism, the release of BBR was governed by a diffusion process, where the diffusion rate was higher than the polymer relaxation [43]. Based on the *R*^2^ values shown in Table 1, the release data of the BBR NPs/PLA nanofiber scaffold was simultaneously well described by the Higuchi and Ritger–Peppas models, suggesting that BBR NPs release was mainly controlled by a diffusion mechanism. However, the value of n determined by the Ritger–Peppas model was in the range of 0.5 and 1.0, which means that the BBR NPs release mechanism could be represented by a non-Fickian diffusion. In other words, the release of BBR NPs was not only based on diffusion but also involved other processes, such as dissolution or degradation of BBR NPs largely concentrated on the surface of the BBR NPs/PLA nanofiber scaffold [43]. In conclusion, the physiochemical properties of the BBR drug greatly affected the distribution of BBR on the PLA nanofibers, subsequently resulting in different BBR release profiles and mechanisms.

**Table 2:** Parameters determined by fitting BBR release data to four different mathematical models.

Mathematical model	Zero order	First order	Higuchi	Ritger–Peppas

BBR/PLA nanofiber scaffold	*K*_0_ = 0.0145 *R*^2^ = 0.9071	*K*_1_ = 0.0836 *R*^2^ = 0.7516	*K*_H_ = 14.69 *R*^2^ = 0.8959	*K*_R_ = 2.1457 *n* = 0.1703 *R*^2^ = 0.9374
BBR NPs/PLA nanofiber scaffold	*K*_0_ = 0.0099 *R*^2^ = 0.8385	*K*_1_ = 0.0164 *R*^2^ = 0.7075	*K*_H_ = 10.623 *R*^2^ = 0.9295	*K*_R_ = 0.4278 *n* = 0.6616 *R*^2^ = 0.918

**Figure 5:**
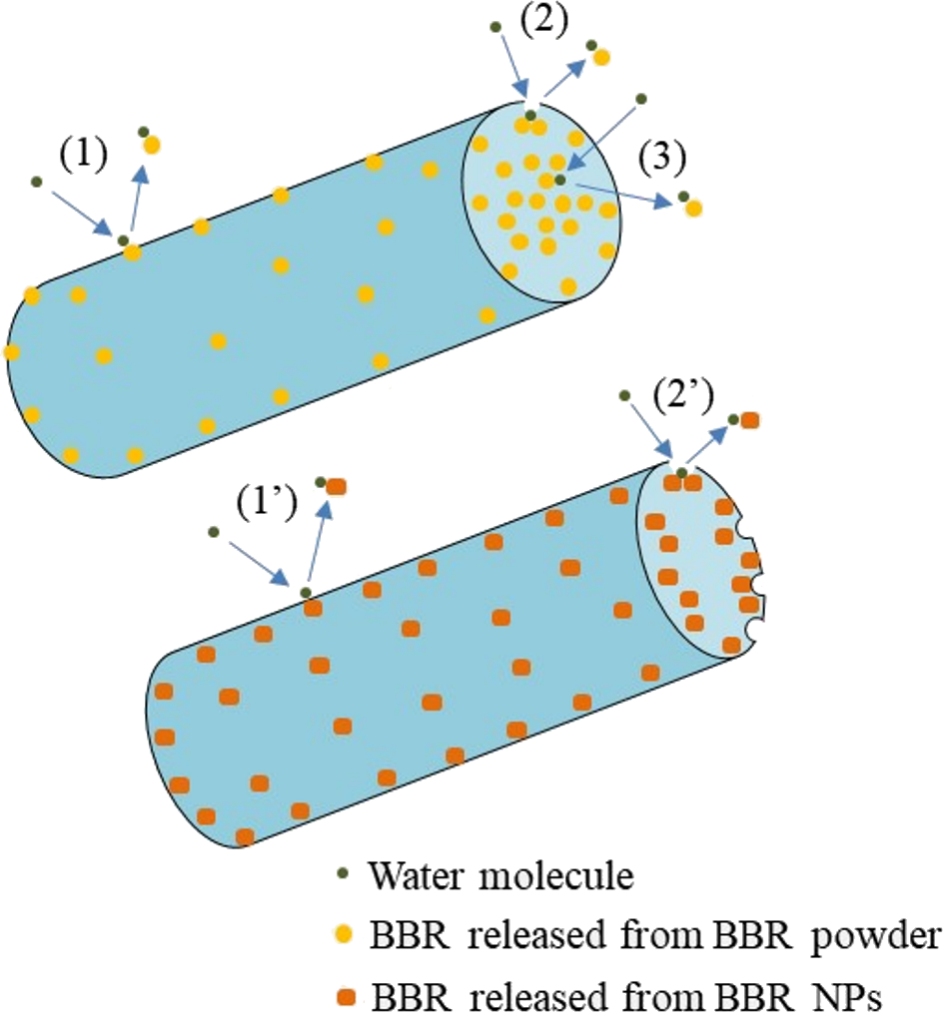
Proposed mechanism of BBR release from BBR/PLA and BBR NPs/PLA nanofiber scaffolds.

The mechanism of BBR release from BBR/PLA and BBR NPs/PLA nanofiber scaffolds was proposed in Figure 5. Since the degradation of PLA nanofibers is a long-term process (over a week) in buffer solution [17], the release of BBR from PLA nanofiber scaffold might be mainly dominated by the distribution of BBR in the nanofibers and the fiber wettability. In the case of hydrophobic BBR dispersed in hydrophobic PLA nanofibers, the release of BBR could take place in three main steps: (1) water molecules diffused from the aqueous medium onto the surface of nanofibers, dissolved the BBR molecules embedded on the surface of nanofibers, and then the dissolved BBR molecules were diffused into the medium. This step took a long time due to the high hydrophobicity of BBR/PLA nanofibers, causing a lag time in the initial stage of release. (2) When the PLA nanofibers were wetted, water molecules penetrated into the nanofibers, resulting in a high release of BBR by diffusion. (3) The BBR molecules in the core of PLA nanofibers slowly diffused out over a prolonged time. Meanwhile, the mechanism of BBR release from BBR NPs/PLA nanofiber scaffolds occurred in two main steps: (1’) water molecules from the aqueous medium quickly diffused and dissolved the BBR molecules embedded on the surface of nanofibers due to their higher hydrophilicity. (2’) The BBR molecules were continuously released thanks to the high concentration of BBR located near the surface of PLA nanofibers. As a result, pores could be formed in the nanofiber matrix, which could allow the gradual diffusion of BBR inside the nanofibers to the aqueous medium.

### Antibacterial performance of BBR-loaded nanofiber scaffolds

**Figure 6:**
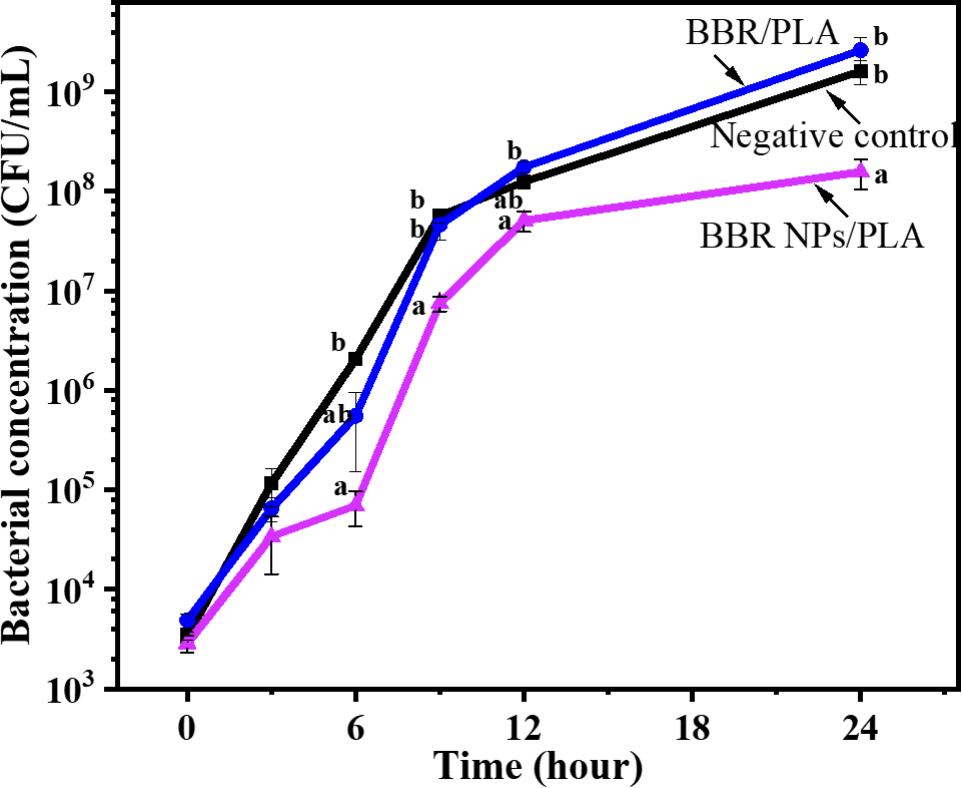
The growth curves of MRSA incubated in nutrient broth (negative control) and treated with BBR/PLA and BBR NPs/PLA nanofiber scaffolds. Different letters indicate significant differences (*p* < 0.05) between groups, whereas the same letter denotes that the differences between groups are nonsignificant (*p* > 0.05). Statistical results were obtained from one-way analysis of variance (ANOVA).

To evaluate the antibacterial efficiency of BBR-loaded nanofiber scaffolds in the relationship with their BBR release profiles, the antibacterial test of these scaffolds against MRSA was performed during 24 h. The antibacterial activity was accessed by the bacterial concentration in the incubation solutions at each time interval corresponding to the amount of released BBR. Figure 6 and Supporting Information File 2 present the negative control and the antibacterial effectiveness of BBR/PLA and BBR NPs/PLA nanofiber scaffolds against MRSA at each time interval. The growth curve of MRSA incubated in the nutrient broth during 24 h shows two distinct phases of bacterial growth, which are exponential and stationary phases. The exponential phase occurred in the first 12 h when the cell numbers were doubled after each generation time. After that, the stationary phase was reached when the number of growth cells was almost equal to that of dead cells. The proliferation of MRSA incubated in the nutrient broth with the presence of the BBR/PLA nanofiber scaffold also exhibited these two phases. The weak inhibitory effect of the BBR/PLA nanofiber scaffold against MRSA was observed over the period of 3 and 9 h. This could be explained by the small amount of BBR released from the BBR/PLA nanofiber scaffold during the first 12 h, as mentioned in the drug-release results. Meanwhile, a notable decrease in MRSA cell growth for 24 h was clearly achieved when MRSA was treated with the BBR NPs/PLA nanofiber scaffold. In addition, the number of MRSA cells was not significantly different between 12 and 24 h, indicating that the stationary phase was reached faster due to the inhibitory activity of the BBR NPs/PLA nanofiber scaffold. It is a fact that the BBR NPs/PLA nanofiber scaffold could sustainably release a high amount of BBR (75%) during 24 h, resulting in the boosted antibacterial effectiveness of the scaffold. A previous study reported that BBR exhibited excellent antibacterial activity against MRSA by damaging the cell wall structure and membrane integrity and further changing the cell morphology in the concentration range of 64–256 mg/L [44]. Recently, Wu et al. [45] proposed a novel orientation on the antibacterial mechanism of BBR against a standard strain *Staphylococcus aureus*, whereby BBR inhibits the synthesis of the cell wall and an aromatic amino acid induces oxidative damage and decreases stress resistance. Besides, BBR was found to inhibit MRSA biofilm formation with the concentration in the range of 1–64 mg/L [46]. In our study, the concentration of BBR released from BBR NPs/PLA nanofiber scaffolds after 6, 12, and 24 h was 87.8, 106.0, and 150.5 mg/L, respectively, which are in the BBR concentration range, leading to an inhibitory effect against MRSA similarly to the aforementioned studies.

### Cytotoxicity of BBR NPs/PLA nanofiber scaffolds

**Figure 7:**
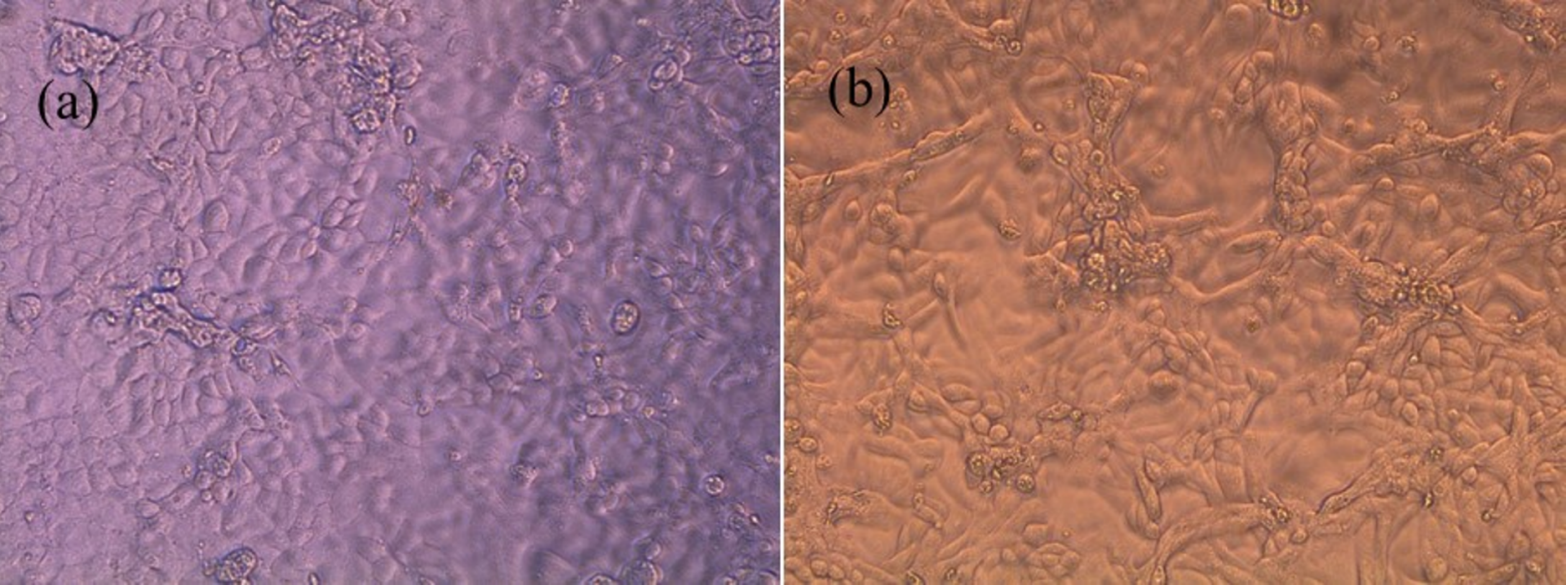
Morphology of (a) control MA-104 cells and (b) MA-104 cells treated with BBR NPs/PLA nanofiber scaffold after 120 h of incubation.

Microbiological associates-104 (MA-104) cell monolayers were cultured with the BBR NPs/PLA nanofiber scaffold, which was cut and fitted to the bottom of the wells of a plastic 96-well plate filled with DMEM. The cells were incubated for 120 h and photographed under a microscopy at each time interval. Figure 7 shows the microscopic examination of MA-104 cells incubated with and without the presence of BBR NPs/PLA nanofiber scaffolds for 120 h. The normal MA-104 monolayers (Figure 7a) homogeneously grew with regular dimensions in a polygonal shape. It can be observed in the control sample that there were a few cells shrunk to a round shape and nearly dead as a result of spontaneous cells degeneration over time. The cells treated with the BBR NPs/PLA nanofiber scaffold (Figure 7b) had a similar cell morphology, suggesting that this scaffold did not exhibit cytotoxic activity against MA-104 cells. Therefore, it is proposed that the BBR NPs/PLA nanofiber scaffold can be a potential candidate for broad biomedical applications, such as wound dressing, drug delivery, and tissue engineering.

## Conclusion

PLA nanofiber scaffolds loaded with BBR powder and BBR NPs were fabricated by electrospinning technique. The average diameter of BBR/PLA and BBR NPs/PLA nanofibers were 351 ± 65 and 356 ± 98 nm, respectively. The chemical characteristics, BBR dispersion into the nanofibers, and fiber wettability of these scaffolds depended on the compatibility of the BBR drug and PLA polymer. The poor compatibility of hydrophilic BBR NPs and hydrophobic PLA resulted in a higher concentration of BBR located on the surface of nanofibers and lower water contact angle value of the scaffold compared to that of the scaffold prepared by the blend of hydrophobic BBR powder and hydrophobic PLA. Consequently, the PLA nanofiber scaffold loaded with BBR NPs gradually released a maximum of 93% of BBR during 64 h and effectively inhibited the proliferation of MRSA during 24 h. Meanwhile, the BBR concentration released from the BBR/PLA nanofiber scaffold during the first 24 h did not reach the minimum inhibition concentration for MRSA. The release of BBR from PLA nanofiber scaffolds was best fit with Ritger–Peppas models, suggesting that BBR release was mainly controlled by a diffusion mechanism. Additionally, the BBR NPs/PLA nanofiber scaffold did not exhibit cytotoxic activity against MA-104 monolayer cells. Different BBR release profiles reported in this study can be suitable design for different applications requiring a certain range of therapeutic concentrations of BBR.

## Experimental

### Materials

Polylactic acid pellets (*M*_w_ of 50,000, purity > 98%) were purchased from Total Corbion (Netherlands). *N*,*N*-Dimethylformamide (≥99.5%) and dichloromethane (>98%) were supplied by Xilong Scientific Co., Ltd., China. Berberine chloride powder (purity > 99%, pharmaceutical primary standard) was commercially obtained from Sigma-Aldrich, Singapore. Nutrient broths were provided by Titan Biotech, India. Bi-distilled water was used to prepare all solutions. All the chemicals were used without any purification.

Methicillin-resistant *Staphylococcus aureus* bacterial strain were isolated from clinical samples of hospitalized patients and stored according to Clinical and Laboratory Standards Institute (CLSI) regulations. The microbiological associates-104 cell line is an epithelial cell from fetal kidney of an African green monkey. The bacterial strain and the MA-104 cell line were provided by the National Institute of Hygiene and Epidemiology, Vietnam.

### Preparation of electrospun PLA, BBR/PLA and BBR NPs/PLA nanofiber scaffolds

Berberine nanoparticles were formed through the antisolvent precipitation process described in our previous report [7]. A 7.0 wt % PLA solution was prepared by dissolving the PLA pellets in a solvent mixture of DCM/DMF with a weight ratio of 80/20 under magnetic stirring for 1 h at 50 °C. After that, the BBR powder and BBR NPs were separately added into the PLA solutions and continuously stirred for 2 h at 40 °C to obtain yellow clear solutions of BBR/PLA and BBR NPs/PLA, respectively. The amount of BBR in these solutions was calculated as 1.0 wt % of PLA composition.

Nanofiber scaffolds of PLA, BBR/PLA, and BBR NPs/PLA were fabricated through the electrospinning of the aforementioned prepared solutions. After the solutions were cooled down to room temperature, they were transferred to a 5 mL syringe with a 22-gauge stainless-steel needle. The needle was linked to a high-voltage power supply (Nano NC, Korea) to generate a 15 kV voltage for the electrospinning process. By using a microinfusion pump, the flow rate of the solution through the needle was maintained at 1.0 mL/h. The distance between the needle tip and the roller collector was fixed at 18 cm. The electrospinning process was conducted for 6 h to obtain the PLA, BBR/PLA, and BBR NPs/PLA nanofiber scaffolds.

### Characterization of prepared scaffolds

The morphology of PLA and BBR-loaded PLA nanofiber scaffolds was observed by a scanning electron microscope (JSM-6510LV). Fiber diameters were measured from the SEM images by using the ImageJ software as an image analysis tool.

Fourier-transform infrared spectroscopy was performed in a Nicolet NEXUS 670 spectrometer. The resulting spectra were recorded in transmission mode in the wavelength range of 500–4000 cm^−1^.

A Raman spectrometer (MacroRAM, Horiba) was used to investigate the chemical characteristics of prepared scaffolds in the wavelength range of 200–3200 cm^−1^. X-ray diffraction measurements of PLA pellets and BBR-loaded PLA nanofiber scaffolds were analyzed with Cu Kα radiation in a 2θ range from 5 to 80° using EQUINOX 5000 – Thermo Scientific X-ray diffractometer.

Static contact angles of the electrospun scaffolds were measured using a Samsung FACED camera (Korea). A drop of bidistilled water was placed on the flat surface of the electrospun scaffold and then a digital image of the drop was taken for measuring the value of the contact angle using an image processing program. All samples were measured at least five times from different locations and the average value was reported.

### In vitro drug-release study

The release of BBR from the scaffolds was performed in a nutrient solution, which was also used for the antibacterial testing in order to assess the relationship between their antibacterial activity with the BBR release profile from these scaffolds. To determine the concentration of BBR in the nutrient solution, a standard calibration curve of UV–vis absorbance versus BBR concentrations was built as follows: 1 mg of BBR powder was dissolved in 1 mL of bidistilled water to obtain a BBR solution stock. Then, this solution was diluted by the nutrient solution in volumetric flasks to make concentrations ranging from 1–200 µg/mL. The absorbance of these BBR solutions was read at 421 nm using a UV–vis spectrophotometer (6850 UV–vis, Jenway).

Electrospun PLA, BBR/PLA, and BBR NPs/PLA nanofiber scaffolds were employed for drug-release tests. The scaffolds were cut into a rectangular shape with a PLA weight of 0.1 g in all samples. Each sample was put in a 10 mL bottle containing 5 mL of nutrient solution. After that, the bottles were shaken using a PTR-35 Vertical Multi-function shaker at room temperature with constant agitation at 40 rpm. At each time interval, 2 mL of each solution was withdrawn, the UV–vis absorbance at 421 nm was measured, and then the amount of BBR release based on the standard calibration curve was calculated. The percentage of released BBR at each time interval was calculated by Equation 1. All the experiments were repeated three times.


[1] $$\text{Percentage of BBR release }\left(\%\right)=\frac{M_{\text{t}}}{M_{\text{m}}}\times 100\%$$

In Equation 1, *M*_t_ (mg) is the weight of BBR released at each time interval and *M*_m_ (mg) is the weight of BBR incorporated in the scaffold.

### Mathematical models

In order to distinguish the mechanism of BBR released from BBR/PLA and from BBR NPs/PLA nanofiber scaffolds, the data of the experimental BBR release were described by four kinetic models, including the zero-order model, first-order model, Higuchi model, and Ritger–Peppas model (Supporting Information File 1).

### Antibacterial test

The antibacterial activity of BBR/PLA and BBR NPs/PLA nanofiber scaffolds was tested against MRSA (Gram-positive bacteria). The antibacterial tests were performed in a sterilized 20 mL glass tube containing bacterial solutions with a concentration of approx. 3 × 10^3^ colony-forming units (CFU)/mL in nutrient broth. BBR/PLA and BBR NPs/PLA nanofiber scaffolds were cut into rectangles with a PLA weight of 0.1 g and then put into the test tubes. For a negative control, one tube was retained without any scaffold sample. Subsequently, all the test tubes were statically incubated at 37 °C for 24 h.

The proliferation of bacteria during 24 h of incubation with and without samples was evaluated by counting the bacterial colonies growing on the agar surface. Briefly, at each incubation time interval (0, 3, 6, 9, 12, 24 h), the solutions in each test tube were diluted many times in physiological saline. A volume of 100 µL of the initial and diluted solutions was spread onto the agar surface in plastic Petri discs and statically incubated at 37 °C for 24 h. Finally, the concentration of bacteria in inoculated solutions was calculated based on the number of bacterial colonies.

### Test of cytotoxicity of BBR NPs/PLA nanofiber scaffolds

The cell culture medium was prepared by mixing 150 mL of Dulbecco’s modified eagle medium (DMEM) with 600 μL of 0.5 mg/mL trypsin in a Schott bottle. MA-104 cells were distributed into the wells of a 96-well plate in DMEM supplemented with 10% fetal bovine serum. The BBR NPs/PLA nanofiber scaffold was cut into circles with a diameter of 6 mm and sterilized with ultraviolet light for 12 h. Then these cut scaffolds were submerged in the cell culture medium in the 96-well plate and incubated for 120 h at 37 °C in an atmosphere of 5% CO_2_. The morphology of the cultured cells was monitored with an inverted microscope every 24 h.

### Statistical analysis

The data were reported as mean values ± standard deviations. Statistical analysis of antibacterial data was performed using one-way analysis of variance (ANOVA). A *p*-value of less than 0.05 was considered statistically significant.

## Supporting Information

File 1 Mathematical models.

File 2 Photographs of agar plates inoculated with MRSA treated with BBR/PLA and BBR NPs/PLA nanofiber scaffolds and negative control.

## Data Availability

All data that supports the findings of this study is available in the published article and/or the supporting information to this article.
