# Supplementary material for: Berberine-loaded polylactic acid nanofiber scaffold as a drug delivery system: The relationship between chemical characteristics, drug-release behavior, and antibacterial efficiency
Source: Beilstein J Nanotechnol. 2024 Jan 12;15:71–82. doi: 10.3762/bjnano.15.7 (PMC10790648; doi:10.3762/bjnano.15.7)
Supplement: File 1 — Mathematical models. [file Beilstein_J_Nanotechnol-15-71-s001.pdf]

BEILSTEIN JOURNAL OF NANOTECHNOLOGY

Supporting Information

for

Berberine-loaded polylactic acid nanofiber scaffold as a drug delivery system: The relationship between chemical characteristics, drug-release behavior, and antibacterial efficiency

Le Thi Le, Hue Thi Nguyen, Liem Thanh Nguyen, Huy Quang Tran
and Thuy Thi Thu Nguyen

Beilstein J. Nanotechnol. **2024,** *15,* 71–82. doi:10.3762/bjnano.15.7

Mathematical models

License and Terms: This is a supporting information file under the terms of the Creative Commons Attribution License (https://creativecommons.org/licenses/by/4.0). Please note that the reuse, redistribution and reproduction in particular requires that the author(s) and source are credited and that individual graphics may be subject to special legal provisions.
The license is subject to the *Beilstein Journal of Nanotechnology* terms and conditions: (https://www.beilstein-journals.org/bjnano/terms)

1. Mathematical models

In order to distinguish the release mechanism of BBR from BBR/PLA and BBR NPs/PLA nanofiber scaffolds, the experimental data of BBR release were described by four kinetic models, including the zero-order model, first-order model, Higuchi model, and Ritger–Peppas model.

1.1. The zero-order model

The zero-order model is described in Equation (2):

$$\frac{C_t}{C_\infty} = K_0 t, \tag{2}$$

where C_∞ and C_t are the concentration of drug release at infinite time and at time t (hours), respectively. K_0 is the zero-order release constant. This model stands for a drug release system in which the rate of drug release is not dependent on concentration.

1.2. The first-order model

The first-order model is described in Equation (3):

$$\frac{C_t}{C_\infty} = 1 - e^{K_1 t}, \tag{3}$$

where K_1 is the first-order constant. This model expresses a drug release system in which the rate of drug release is concentration dependent.

1.3. Higuchi model

The Higuchi model is shown in Equation (4):

$$\frac{C_t}{C_\infty} = K_H t^{\frac{1}{2}}, \tag{4}$$

where K_H is the Higuchi dissolution constant. This model is used to study the release mechanism of water-soluble and low-soluble drugs loaded into solid matrices. The drug release according to the Higuchi model follows a diffusion process based on Fick's law.

1.4. Ritger-Peppas model

The Ritger-Peppas model is represented in Equation (5):

$$\frac{C_t}{C_\infty} = K_R t^n, \tag{5}$$

where K_R is the Ritger–Peppas kinetic constant, n is the diffusion exponent indicating the release mechanism of drug from polymer matrices. When $n \leq 0.5$, the release mechanism follows a Fickian diffusion. $0.5 < n < 1.0$ indicates non-Fickian diffusion, including other mechanisms associated with the diffusion. A value of $n = 1$ represents zero-order release, which is independent of time.

2. Results

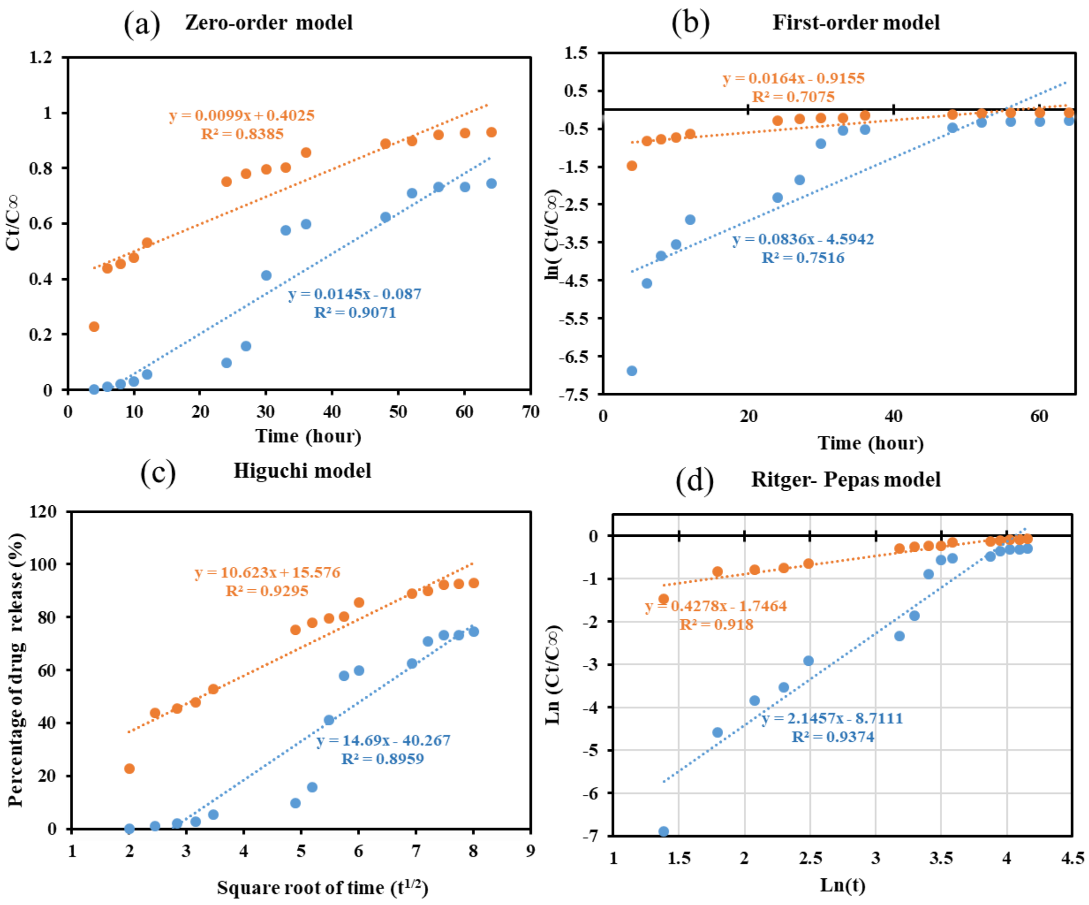

Figure S1: The equations and parameters determined by fitting BBR release data to mathematical models of (a) the zero-order model, (b) the first-order model, (c) the Higuchi model, and (d) the Ritger–Pepas model. The blue curves represent the BBR/PLA nanofiber scaffold and the orange curves represent the BBR NPs/PLA nanofiber scaffold.
