# Supplementary material for: Berberine-loaded polylactic acid nanofiber scaffold as a drug delivery system: The relationship between chemical characteristics, drug-release behavior, and antibacterial efficiency
Source: Beilstein J Nanotechnol. 2024 Jan 12;15:71–82. doi: 10.3762/bjnano.15.7 (PMC10790648; doi:10.3762/bjnano.15.7)
Supplement: File 2 — Photographs of agar plates inoculated with MRSA treated with BBR/PLA and BBR NPs/PLA nanofiber scaffolds and negative control. [file Beilstein_J_Nanotechnol-15-71-s002.pdf]

BEILSTEIN JOURNAL OF NANOTECHNOLOGY

Supporting Information

for

Berberine-loaded polylactic acid nanofiber scaffold as a drug delivery system: The relationship between chemical characteristics, drug-release behavior, and antibacterial efficiency

Le Thi Le, Hue Thi Nguyen, Liem Thanh Nguyen, Huy Quang Tran
and Thuy Thi Thu Nguyen

Beilstein J. Nanotechnol. **2024,** *15,* 71–82. doi:10.3762/bjnano.15.7

Photographs of agar plates inoculated with MRSA treated with BBR/PLA and BBR NPs/PLA nanofiber scaffolds and negative control

License and Terms: This is a supporting information file under the terms of the Creative Commons Attribution License (https://creativecommons.org/licenses/by/4.0). Please note that the reuse, redistribution and reproduction in particular requires that the author(s) and source are credited and that individual graphics may be subject to special legal provisions.
The license is subject to the *Beilstein Journal of Nanotechnology* terms and conditions: (https://www.beilstein-journals.org/bjnano/terms)

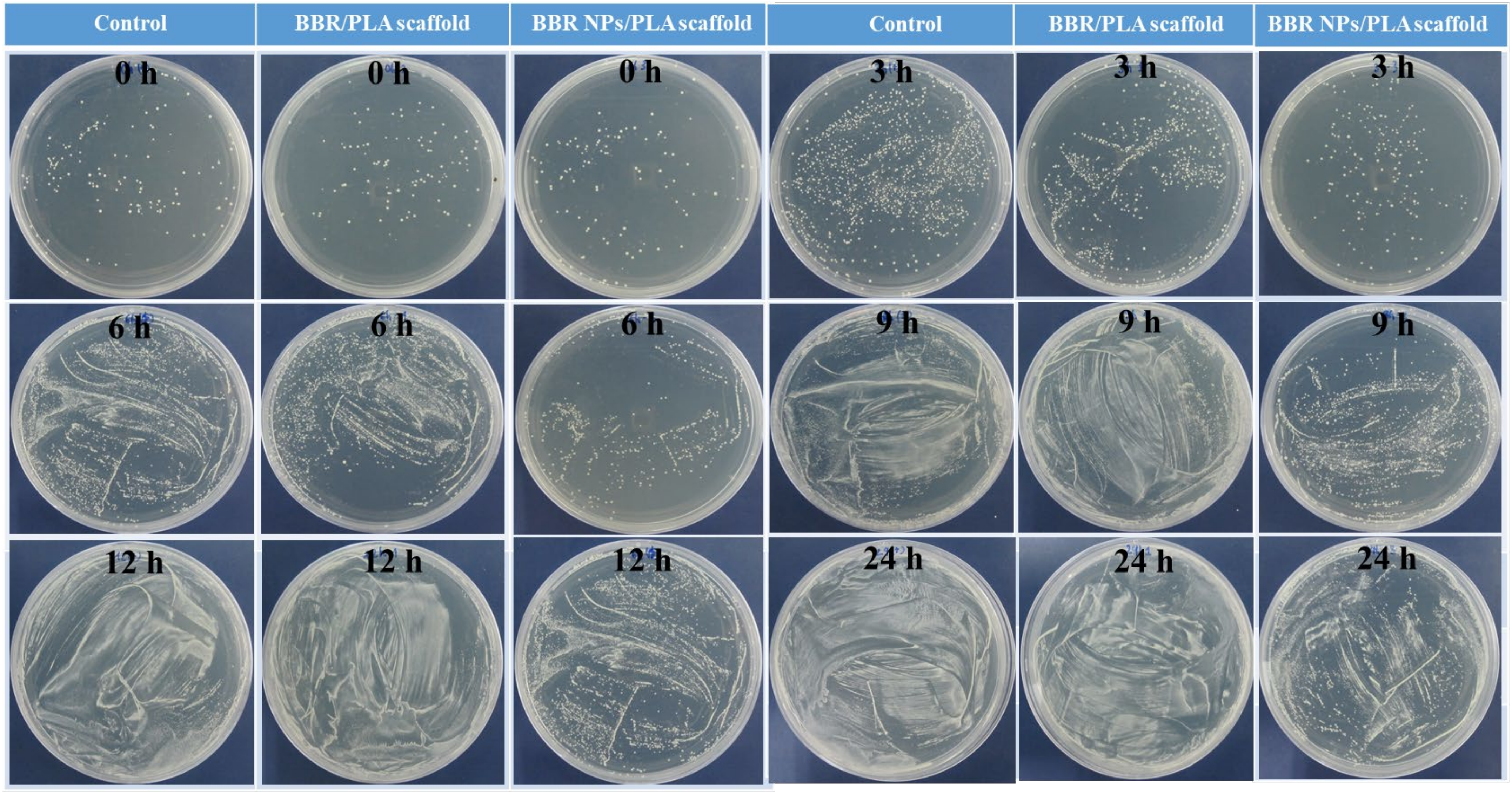
Control
BBR/PLA scaffold
BBR NPs/PLA scaffold
Control
BBR/PLA scaffold
BBR NPs/PLA scaffold
0 h
0 h
0 h
3 h
3 h
3 h
6 h
6 h
6 h
9 h
9 h
9 h
12 h
12 h
12 h
24 h
24 h
24 h
